# Sex-Specific Effect of Serum Lipids and Body Mass Index on Psychotic Symptoms, a Cross-Sectional Study of First-Episode Psychosis Patients

**DOI:** 10.3389/fpsyt.2021.723158

**Published:** 2021-10-21

**Authors:** Priyanthi B. Gjerde, Carmen E. Simonsen, Trine V. Lagerberg, Nils Eiel Steen, Ole A. Andreassen, Vidar M. Steen, Ingrid Melle

**Affiliations:** ^1^Norwegian Centre for Mental Disorders Research, Department of Clinical Science, University of Bergen, Bergen, Norway; ^2^Dr. Einar Martens Research Group for Biological Psychiatry, Department of Medical Genetics, Haukeland University Hospital, Bergen, Norway; ^3^Research Unit for General Practice, NORCE Norwegian Research Centre, Bergen, Norway; ^4^Norwegian Centre for Mental Disorders Research, Oslo University Hospital, Oslo, Norway; ^5^Institute of Clinical Medicine, University of Oslo, Oslo, Norway

**Keywords:** schizophrenia, psychosis, gender, serum lipids, BMI, clinical outcome

## Abstract

**Background:** Schizophrenia is a disorder with considerable heterogeneity in course and outcomes, which is in part related to the patients' sex. Studies report a link between serum lipids, body mass index (BMI), and therapeutic response. However, the role of sex in these relationships is poorly understood. In a cross-sectional sample of first-episode psychosis (FEP) patients, we investigated if the relationship between serum lipid levels (total cholesterol, HDL-C, LDL-C, and triglycerides), BMI, and symptoms differs between the sexes.

**Methods:** We included 435 FEP patients (males: *N* = 283, 65%) from the ongoing Thematically Organized Psychosis (TOP) study. Data on clinical status, antipsychotics, lifestyle, serum lipid levels, and BMI were obtained. The Positive and Negative Syndrome Scale (PANSS) and the Calgary Depression Scale for Schizophrenia (CDSS) were used to assess psychotic and depressive symptoms. General linear models were employed to examine the relationship between metabolic variables and symptomatology.

**Results:** We observed a female-specific association between serum HDL-C levels and negative symptoms (*B* = −2.24, *p* = 0.03) and between triglycerides levels (*B* = 1.48, *p* = 0.04) and BMI (*B* = 0.27, *p* = 0.001) with depressive symptoms. When controlling for BMI, only the association between serum HDL-C levels and negative symptoms remained significant. Moreover, the HDL-C and BMI associations remained significant after controlling for demography, lifestyle, and illness-related factors.

**Conclusion:** We found a relationship between metabolic factors and psychiatric symptoms in FEP patients that was sex-dependent.

## Introduction

Sex differences are widely reported in schizophrenia and related psychoses (1–5). Males exhibit earlier onset, poorer premorbid functioning, higher propensity to negative symptoms, and poorer treatment outcomes (1, 3). Females, on the other hand, report more affective symptoms, and often demonstrate better psychosocial functioning and a better prognosis after drug treatment (4–6).

Accumulating studies have further revealed sex differences for metabolic measures (7–12). Although such sex-related differences are also observed in the general population, recent reports conclude that sex differences are more extensive in psychotic patients (10, 13).

While antipsychotic drugs are cornerstones in the treatment of psychosis, it is widely recognized that such medications contribute to high rates of metabolic co-morbidity, including metabolic syndrome, weight gain, and dyslipidemia (7, 14, 15). The prevalence and nature of these metabolic disturbances seem to differ between males and females (8, 9, 16). The Clinical Antipsychotic Trials of Intervention Effectiveness (CATIE) study found that the prevalence of metabolic syndrome was much higher in antipsychotic-treated female patients than male patients (51.5 vs. 36%) (10). Female sex has also been related to more extensive weight gain following antipsychotic drug treatment (17), whereas male sex has been linked to lower HDL-C and higher triglyceride (TG) levels following drug treatment (18, 19). One proposed mechanism underlying these sex differences in metabolic co-morbidities is the influence of sex hormones on regulating food intake, energy expenditure, and drug metabolism (20–22).

Antipsychotic-induced activation of lipid biosynthesis has been proposed as one of the mechanisms underlying the metabolic side-effects (23–25). Intriguingly, studies in cell cultures and animal models have indicated that metabolic disturbances might be linked to the therapeutic efficiency of antipsychotic drugs (24–26). In line with these findings, studies of antipsychotic-treated schizophrenia patients demonstrate a relationship between weight gain, serum lipid changes, and improvement in psychosis (27–36).

While research on possible links between metabolic measures and psychosis during antipsychotic drug treatment is growing, only limited attention has been given to studying the influence of sex on these relationships. The results of these few studies have moreover been inconsistent (37–41). For example, Hung et al. (38) found that risperidone-induced weight gain correlated closely with clinical response in females but not in males. Wei et al. (42), on the other hand, reported negative associations of body mass index (BMI) with negative symptoms and general psychopathology in male patients only. Still, others report no sex differences in the relationship between weight gain and therapeutic efficacy (37, 40). Most previous studies have focused on chronic schizophrenia patients, where it may be challenging to disentangle sex-related effects from illness chronicity. Many studies have also lacked to examine potential influences of poor lifestyle habits, which have been shown to differ between the sexes (1). Finally, studies have yet to examine whether the relationship between serum lipids and psychosis during antipsychotic drug treatment differs between males and females.

Hence, the objective of the present study was to expand on prior research of sex differences in psychosis while investigating if the sex of the patient affects the relationship between serum lipids, BMI, and psychotic symptoms in antipsychotic-treated first-episode psychosis (FEP) patients. As studies have demonstrated negative associations between weight/BMI, serum lipid levels (mostly for TG and HDL-C levels) and psychotic symptoms during antipsychotic drug treatment, as well as sex differences for each of these measures, we hypothesized that in our sample of antipsychotic-treated FEP patients, we would find (i) a sex-specific negative association between BMI and symptoms of psychosis, and (ii) a sex-specific negative association between serum lipid levels, especially for TG and HDL-C levels, and symptoms of psychosis.

## Materials and Methods

### The Sample

The present study was cross-sectional in design, consisting of 435 FEP patients recruited at their first treatment for a psychotic disorder from psychiatric units in Oslo, Norway, as part of the larger Thematically Organized Psychosis (TOP) study (http://www.med.uio.no/norment/english/). The study sample overlaps with a FEP cohort used to examine metabolic side effects during antipsychotic drug treatment (*n* = 132) including these patients in the current sample and analyses [for details, see Gjerde et al. (32)]. Inclusion criteria for the TOP study were age between 18 and 65 years and having a diagnosis within a broad schizophrenia spectrum psychosis according to DSM-IV. Participants with an IQ <70, significant head injury, or neurological disorders were excluded. In addition to the general criteria, the specific criterium for the FEP sample was that the patient had not previously received adequate antipsychotic drug treatment for their psychotic disorder. Adequate treatment was defined as antipsychotic medication in doses over 1 Defined Daily Dosage (DDD) for >12 weeks or shorter if this treatment was followed by symptomatic remission [for details, see Gjerde et al. (32)].

All participants gave written informed consent before inclusion in the study, and the Regional Ethics Committee and The Norwegian Data Inspectorate approved the study.

### Clinical Assessments

Demographic and clinical data were obtained through structured interviews and from hospital records. The Structural Clinical Interview for DSM-IV (SCID) (43) was used to ascertain the diagnosis. The Positive and Negative Syndrome Scale (PANSS) (44) was used to assess psychotic symptoms. In the current study, the five-factor PANSS model by Wallwork et al. (45), found to be suitable for FEP samples (46), was used with an emphasis on the positive factor (items P1, P3, P5, and G9) and the negative factor (items N1, N2, N3, N4, N6, and G7). The Calgary Depression Scale for Schizophrenia (CDSS) (47) was applied for depressive symptoms. The Alcohol Use Disorders Identification Test (AUDIT) (48) and the Drug Use Disorders Identification Test (DUDIT) (49) were used to measure alcohol use and illicit drug use. Diet and exercise habits were assessed at inclusion by interviews. The patients were asked to describe their eating habits as healthy, moderately healthy or unhealthy and their exercise habits as light, moderate or hard (32).

### Use of Antipsychotics and Other Medications

Patient interviews complemented by medical charts were used to acquire information about previous and current use of antipsychotics. Compliance was assessed by measuring antipsychotic drug levels in serum samples analyzed at the Department of Clinical Pharmacology, St. Olav University Hospital, Trondheim, Norway [see (50) for details].

Antipsychotic drug treatment was dichotomized into antipsychotic medicated and non-medicated. In addition, we examined putative differences concerning antipsychotic monotherapy vs. polypharmacy (more than one antipsychotic medication), as well as potential differential effects of monotherapy with second-generation antipsychotic (SGA) drugs based on their metabolic profiles (15, 51, 52): metabolically potent SGA (focusing on olanzapine), metabolically intermediate SGA (quetiapine and risperidone), and metabolically neutral SGA (aripiprazole, amisulpride, ziprasidone, sertindole).

We did not investigate differences between first-generation (FGA) and SGA medications due to a low number of FGA users (*n* = 10).

Other medications in our FEP sample included four patients on lithium, two on antiepileptic drugs (lamotrigine and clonazepam), and 124 on antidepressants. Of note, none of the participants used medications for diabetes, hypertension, dyslipidemia (statins and fibrates), or thyroid abnormalities.

### Serum Lipid and BMI Measurements

Height and weight were measured using hospital quality weight and height scales under standardized settings. BMI (kg/m^2^) was calculated as weight (in kg) divided by height (in meters) squared. Fasting blood was drawn and processed to analyze serum levels of lipids at the Department of Medical Biochemistry at Oslo University Hospital, using standard enzymatic methods from Roche Diagnostics Norge AS (Oslo, Norway). The following lipid parameters were measured: total cholesterol, low-density lipoprotein (LDL-C), high-density lipoprotein (HDL-C), and triglycerides (TG) [for details, see Gjerde et al. (32)]. Patients that were on any particular antipsychotic drug treatment at inclusion had used it for 3–7 weeks when the BMI and serum lipids were assessed.

### Statistical Analysis

Statistical analyses were performed using SPSS version 25. For comparison of demographic and clinical characteristics related to the sex of the patient, chi-square tests for categorical variables and *t*-tests for continuous variables were used. A significance level of *p* < 0.05 (two-tailed) was applied.

Regression analyses using General linear models (GLMs) controlling for age, sex, and antipsychotic usage (antipsychotic users vs. non-users) were employed to examine the relationship between metabolic variables and outcome measures (positive and negative PANSS factors and CDSS total score). Also included in these primary analyses was a sex × metabolic parameter (BMI/total cholesterol/HDL-C/LDL-C/TG, respectively) interaction term for assessing the influence of sex. All outcome measures were investigated in separate models along with each metabolic parameter (BMI/total cholesterol/HDL-C/LDL-C/TG). Preliminary analyses were conducted to ensure assumptions of normality and linearity for the GLMs; no violations of the assumptions were found. Benjamini and Hochberg's procedure with a false discovery rate set at 0.25 was applied for multiple testing.

In *post-hoc* analyses, we further examined if there were any correlations between serum lipid levels and BMI by using Spearman's rho as initial examinations of the relationship between serum lipids and BMI showed that the assumptions of constant variance and linearity were not fully met, but that the metabolic variables were monotonically related. We also examined if any sex-related associations between serum lipids and clinical outcomes (PANSS factors and CDSS scores) were confounded by BMI (using similar GLM as in the main analyses). Moreover, we investigated if significant group difference found in the group-wise analyses in demography, lifestyle factors (smoking, alcohol use, diet, and exercise), illness factors (DUP, hospitalization, diagnosis), prior antipsychotic drug exposure (drug-naïve vs. non-naïve) and current antipsychotic treatment regime (monotherapy vs. polypharmacy, monotherapy with a metabolically potent SGA and intermediate SGA vs. neutral SGA), affected our main results. Finally, as there was a relatively large proportion of antidepressant users (28.5%), and due to a possible effect of these medications on metabolic measures and depressive symptoms, we also controlled for treatment with antidepressants (dichotomized as yes/no) where appropriate.

## Results

### Description of the Sample

Four hundred and thirty-five FEP patients participated in the current study, of whom 283 (65%) were males. The clinical characteristics of the study population according to the sex of the patient are shown in Table 1, while details on prior antipsychotic drug exposure and current antipsychotic drug regime are shown in Supplementary Table 1.

**Table 1:** Group comparison of demographic, lifestyle, illness, and metabolic variables.

	**Males included in the analyses**	***N* (%)/Mean (SD)**	**Females included in the analyses**	***N* (%)/Mean (SD)**	**Chi/*t*-tests**	**df**	***p*-value**
Age (years)	283	26.69 (7.56)	152	27.38 (9.04)	−0.84	433	0.40
Ethnicity (Caucasian)	283	222 (78)	151	129 (85)	2.66	1	0.10
Education (total years)	279	12.5 (2.8)	151	13.0 (2.7)	−1.91	428	0.06
Smoking (daily)	277	160 (57)	152	71 (47)	4.82	1	**0.03**
AUDIT (total scores)	233	8.0 (7.44)	122	6.6 (7.19)	1.62	353	0.11
DUDIT (total scores)	242	5.4 (8.46)	132	3.3 (8.10)	2.31	372	**0.02**
Diet	264		150		0.64	2	0.73
Healthy		166 (59)		100 (66)			
Moderately healthy		83 (29)		43 (28)			
Unhealthy		15 (5)		7 (5)			
Exercise	210		121		4.31	2	0.12
Light		133 (47)		90 (59)			
Moderate		58 (21)		24 (16)			
Hard		19 (7)		7 (5)			
Diagnosis	283		152		21.7	3	** <0.001**
Schizophrenia		168 (59)		69 (45)			
Schizophreniform		17 (6)		17 (11)			
Schizoaffective		13 (5)		24 (16)			
Other psychosis		85 (30)		42 (28)			
Hospitalization	269	128 (45)	134	48 (32)	5.03	1	**0.03**
DUP (weeks)	275	133.25 (238.46)	144	158.69 (228.03)	−1.05	417	0.29
PANSS positive factor	281	10.7 (4.14)	152	10.6 (4.02)	0.35	431	0.73
PANSS negative factor	281	13.8 (5.81)	152	12.3 (5.90)	2.52	431	**0.01**
CDSS (total scores)	270	5.3 (4.25)	142	7.5 (5.22)	−4.59	410	** <0.001**
AP use	283	218 (77)	152	115 (76)	0.10	1	0.75
AP drug-naïve	283	39 (14)	152	26 (17)	0.86	1	0.35
Antidepressant use	283	71 (25)	152	53 (35)	4.64	1	**0.03**
BMI (kg/m^2^)	259	25.6 (4.40)	142	24.4 (5.13)	2.28	399	**0.02**
Total cholesterol (mmol/L)	236	5.02 (1.01)	137	4.96 (1.08)	0.53	371	0.59
HDL-C (mmol/L)	236	1.22 (0.31)	137	1.59 (0.46)	−9.27	371	** <0.001**
LDL-C (mmol/L)	222	3.23 (0.89)	128	2.95 (0.91)	2.75	348	**0.01**
TG (mmol/L)	236	1.53 (1.03)	136	1.10 (0.60)	4.45	370	** <0.001**

*SD, standard deviation; N, number of subjects; %, percentage; df, degrees of freedom; AUDIT, Alcohol Use Disorder Identification Test; DUDIT, Drug Use Disorders Identification Test; DUP, duration of untreated psychosis; PANSS, Positive and Negative Syndrome Scale; CDSS, Calgary Depression Scale for Schizophrenia; AP, antipsychotic use; BMI, body mass index; HDL-C, high-density lipoprotein cholesterol; LDL-C, low-density lipoprotein cholesterol. P-values are obtained from T-tests and Chi-square test. Bold font indicates statistical significance for the T-tests and Chi-square test*.

There were significantly more tobacco smokers and illicit-drug users among male patients than females (*p* = 0.03) and (*p* = 0.02), respectively. There was also a significant difference between the sexes for diagnosis; a larger proportion of males compared to females had schizophrenia, while more females had a schizoaffective diagnosis (*p* < 0.001). Additionally, a significantly higher proportion of males compared to females was hospitalized (*p* = 0.03). Females were also more inclined to receive antidepressants (*p* = 0.03). Of note, there were no significant differences between the sexes for diet, exercise, prior antipsychotic drug exposure (drug-naïve vs. non-naïve) or current antipsychotic drug regime (antipsychotic users vs. non-users, monotherapy vs. polypharmacy; monotherapy with a metabolically potent SGA and intermediate SGA vs. neutral SGA).

Males experienced more negative symptoms (*p* = 0.01), and females more depressive symptoms (*p* < 0.001). No significant sex differences were found for positive psychotic symptoms (Table 1).

### Sex-Related Differences in Metabolic Factors

In comparison with female FEP patients, male patients exhibited higher mean BMI, higher serum LDL-C and TG levels, and lower HDL-C levels (all *p* < 0.05) (Table 1).

### Sex-Related Differences in the Associations Between Metabolic Factors and Clinical Symptoms

The *F*-test for the interaction term sex × HDL-C was significant for negative symptoms [*F*_(2,365)_ = 3.41, *p* = 0.03]. Examining the standard estimates and *p*-values for females and males separately, we found that the association between higher HDL-C levels and less negative symptoms was significant only for females (females: *B* = −2.24, *p* = 0.03 vs. males: *B* = −1.84, *p* = 0.13) (Table 2). Moreover, the *F*-test for the interaction terms sex × TG and sex × BMI were significant for depressive symptoms [*F*_(2,327)_ = 3.64, *p* = 0.03] and [*F*_(2,375)_ = 5.27, *p* = 0.006]. Also, here, the standard estimates and *p*-values for females and males indicated that the associations between higher TG levels and BMI with more depressive symptoms were significant for females only, (for TG: females: *B* = 1.48, *p* = 0.04 vs. males: *B* = 0.53, *p* = 0.09) and (for BMI: females: *B* = 0.27, *p* = 0.001 vs. males: *B* = 0.02, *p* = 0.80), respectively (Table 2).

**Table 2:** Regression analyses examining sex-related association between metabolic measures and psychotic symptoms.

	**BMI**		**Total cholesterol**		**HDL-C**		**LDL-C**		**TG**	
	***F***	**B (SE)**	***F***	**B (SE)**	***F***	**B (SE)**	***F***	**B (SE)**	***F***	**B (SE)**
PANSS positive **factor**	*F*_(2, 393)_ = 1.43, *p* = 0.24	*M*: −0.016 (0.06)	*F*_(2, 365)_ = 0.59, *p* = 0.56	*M*: 0.206 (0.27)	*F*_(2, 365)_ = 1.59, *p* = 0.21	*M*: −1.451 (0.84)	*F*_(2, 342)_ = 0.99, *p* = 0.37	*M*: 0.342 (0.31)	*F*_(2, 364)_ = 1.28, *p* = 0.28	*M*: 0.256 (0.25)
		*F*: 0.112 (0.07)		*F*:−0.236 (0.33)		*F*: −0.35 (0.75)		*F*: −0.33 (0.40)		*F*: 0.717 (0.58)
PANSS negative factor	*F*_(2, 393)_ = 0.70, *p* = 0.50	*M*: 0.083 (0.08)	*F*_(2, 365)_ = 1.43, *p* = 0.24	*M*: 0.634 (0.38)	***F***_**(2, 365)**_ **=** **3.41**, ***p*** **=** **0.03**	M:−1.837 (1.20)	*F*_(2, 342)_ = 2.46, *p* = 0.09	*M*: 0.916 (0.45)	*F*_(2, 364)_ = 2.12, *p* = 0.12	*M*: 0.535 (0.36)
		*F*: 0.059 (0.09)		*F*: 0.213 (0.47)		*F*: −2.238 (1.06)		*F*: 0.538 (0.57)		*F*: 1.174 (0.82)
CDSS	***F***_**(2, 375)**_ **=** **5.27**, ***p*** **=** **0.006**	*M*: 0.017 (0.07)	*F*_(2, 57)_ = 2.49, *p* = 0.09	*M*: 0.656 (0.33)	*F*_(2, 347)_ = 3.33, *p* = 0.06	*M*: −2.041 (1.04)	*F*_(2, 347)_ = 3.31, *p* = 0.06	*M*: 0.742 (0.38)	***F***_**(2, 327)**_ **=** **3.64**, ***p*** **=** **0.03**	*M*: 0.529 (0.32)
		*F*: 0.269 (0.08)		*F*: 0.443 (0.40)		*F*: −1.538 (0.92)		*F*: 0.87 (0.49)		*F*: 1.478 (0.70)

*Only the F-values for the interaction term (sex × metabolic parameter) are listed. The standard beta values in the interaction term are also listed as separate values for males and females.*

*BMI, body mass index; HDL-C, high-density lipoprotein cholesterol; LDL-C, low-density lipoprotein cholesterol; TG, triglycerides; F, f-value; B, regression coefficient; SE, standard error of the regression coefficient; PANSS, Positive and Negative Syndrome Scale; CDSS, Calgary Depression Scale for Schizophrenia. Analyzed with general linear models examining a sex × metabolic parameter (BMI/total cholesterol/HDL-C/LDL-C/TG) interaction controlling for age, sex, and antipsychotic usage (antipsychotic users vs. non-users) group. Bold font indicates statistical significance for the interaction term (sex × metabolic parameter)*.

### *Post-hoc* Analyses of the Main Results Controlling for Lifestyle-, Illness- and Medication-Related Factors

There were significant correlations between all serum lipids and BMI (total cholesterol: *r* = 0.214, *p* < 0.001, HDL-C: *r* = −0.252, *p* < 0.001, LDL-C: *r* = 0.233, *p* < 0.001, TG: *r* = 0.322, *p* < 0.001). While adjusting for BMI did not alter the observed sex-specific relationship between serum HDL-C levels and negative symptoms [*F*_(2, 347)_ = 3.52, *p*= 0.03]; the sex-specific association between serum TG levels and depressive symptoms was no longer significant [*F*_(2, 329)_ = 2.31, *p* = 0.10]. When including variables that were significantly different between the sexes (i.e., hospitalization, tobacco smoking, and illicit drug use), the associations found among the female patients remained statistically significant (Supplementary Table 2). As more males than females had a schizophrenia diagnosis, and studies show that illness severity can influence lifestyle habits, antipsychotic drug use and metabolic measures, we also examined the influence of diagnosis by adding this as a covariate in the *post-hoc* analyses; the associations observed for the female patients remained statistically significant (Supplementary Table 2). Also, when we reran the analyses including only patients with a schizophrenia diagnosis, we found that the sex-specific interactions remained significant. Finally, the sex-specific associations remained significant after controlling in part for prior antipsychotic drug exposure (i.e., drug-naïve vs. non-naïve) and for any differences in current drug regimens (i.e., antipsychotic monotherapy vs. polypharmacy; SGA with different metabolic profiles; antidepressant use) (Supplementary Table 2).

## Discussion

The present cross-sectional study of antipsychotic-treated FEP patients demonstrates a significant negative association between HDL-C and negative symptoms and a positive association between BMI and depressive symptoms in females. These associations remained significant after controlling for potential confounders, including demography, lifestyle, illness- and treatment-related factors. Hence, our results indicate that the sex of the patient may play an important role in delineating a possible link between specific metabolic measures and clinical outcomes.

Our findings of sex-specific association of HDL-C with specific psychosis symptoms during drug treatment add new knowledge to a growing literature on a *general* link between serum lipid levels and psychosis symptomatology (27–32, 35, 53, 54). In a recent 1 year follow-up study with an overlapping FEP sample (*n* = 132), we found an increase in HDL-C levels linked with improvement in negative symptoms independent of concurrent changes in BMI (32). This association was also significant after controlling for lifestyle (e.g., smoking, diet, exercise habits) and antipsychotic use. Due to the limited sample size, we were not able to examine the influence of sex. It is, nevertheless, widely recognized that sex differences exist for metabolic co-morbidities (7–12), in symptom expression and treatment outcome (4–6). Females, in particular, have repeatedly displayed favorable lipid profiles and better treatment response (18, 19). It is, therefore, of particular interest that in the present FEP-study, we found a female-specific association between higher HDL-C levels and less negative symptoms.

Factors such as illness severity (e.g., schizophrenia diagnosis), lifestyle habits and antipsychotic medication use can influence psychosis symptoms and serum lipid levels (14, 15). These factors have also been shown to differ between the sexes (4–6). In the present study, there were significant differences in diagnosis with more males having a schizophrenia diagnosis and being smokers, while no sex differences were found for antipsychotic treatment regime. To ensure that these factors were not unduly influencing the sex-specific relationship between HDL-C and negative symptoms, we controlled for diagnosis, lifestyle- and treatment-related factors in subsequent analyses, only to find that the sex-specific association remained significant. While we acknowledge that the present study design constrains us from making causal inferences, one could speculate as to whether innate factors pertaining to the sex of the patient may mitigate the relationship between serum lipids and therapeutic response.

In addition to the sex-specific link between HDL-C and negative symptoms, the present study demonstrates an association between higher BMI and more depressive symptoms in female FEP patients. The discrepancies between our results with some of those previously reported documenting a beneficial link (38, 41), might be a consequence of differences in the sample composition (FEP vs. chronic schizophrenia), sample sizes (ours was relatively large), and specific symptoms assessed (depressive symptoms vs. general psychopathology). The inconsistencies may also be due to the studies above restricting themselves to specific antipsychotic agents (i.e., clozapine and risperidone). Metabolically potent antipsychotic drugs and many antidepressants have been related to improvements in depressive symptoms but also to excessive weight gain (15, 55). The favorable link observed in the studies described above could, in theory, reflect better medication concordance rather than representing a causal link. Our study could not find confounding effects of different antipsychotic treatment regimens or antidepressant usage on the observed association between BMI and depressive symptoms.

Our results are otherwise in accordance with studies from the general population reporting of higher BMI/weight correlating with more depressive symptoms in females (56, 57), although the directionality of this relationship is uncertain (58). Having a higher BMI may lead to depression or be one of its consequences (58). Geofrey et al. tried to tackle this issue in a birth cohort study of more than 18,500 individuals. They found a unidirectional association where obesity predicted elevated risk of subsequent depression by 34% in females, but depression did not predict obesity (57).

In the current study, we also observed a significant association between TG levels and depressive symptoms among female patients. As previous studies indicate strong correlations between TG levels and BMI (59), it was necessary to disentangle TG's role from BMI. Accordingly, the relationship between serum TG levels and depressive symptoms was no longer significant when controlling for BMI. While correlation analyses cannot determine the directionality and causes of the displayed relationship between BMI and TG levels, one possibility is that abdominal adipose tissue cells may produce free fatty acids, thereby increasing serum TG levels (60, 61). Interestingly, studies show that this fatty acid producing effect is more prominent in females (61, 62). Additional studies have further suggested that intrinsic modifying factors may allow for more pronounced medication effects on BMI amongst females (22).

Our findings of a sex-based difference in the relationship between serum HDL-C levels, BMI, and symptoms of psychosis is likely to be multifactorial, where one possible factor is the involvement of sex hormones. Reviews by Lizcano and Mauvais highlight the role of estrogen in regulating adipose tissue distribution, appetite, energy regulation, and fat metabolism (63, 64), which could explain the displayed sex differences for metabolic measures during antipsychotic drug treatment. There are also strong indications of estrogen being protective against psychotic symptoms, predominantly negative- and depressive symptoms (3, 65, 66).

Estrogen receptors are widely distributed in the brain and hold important physiological roles in neurogenesis, synaptic plasticity and myelination, and are, thus, critical for brain homeostasis (67–70). Impairments in myelination and synaptic communication have likewise been established in the pathophysiology of schizophrenia and related psychosis (71–73), and studies have further demonstrated sex difference in white matter abnormalities and its association with symptoms in schizophrenia (74). Given that antipsychotic drugs stimulate lipid biosynthesis with possible effects on myelin-related structures (24, 26, 75, 76) and that estrogen regulates the synthesis of structural proteins essential for cholesterol formation (77), it is plausible that these two pathways interact to improve symptoms of psychosis. Supporting such an interpretation is that impairments in estrogen- and lipid metabolism have been implicated in the development of and in predicting the severity of schizophrenia (3, 11, 78–83). Further support of an estrogen-cholesterol interaction comes from studies of other neurological conditions with a similar sex bias and abnormal cholesterol metabolism, such as Alzheimer's disease (84) and multiple sclerosis (85).

## Limitations

Our data on serum lipids, BMI, and clinical characteristics are cross-sectional and do not allow us to make inferences about causality. Still, as our sample size was relatively large our findings could provide a useful springboard to further research. The lack of data on serum estrogen and other sex hormones is also an important constraint as e.g., higher levels of estrogen has been related to favorable serum lipid levels and to less illness severity (3, 63). With that said, it may be difficult to fully understand the effects of estrogen and other sex hormones in a clinical setting as there are most likely complex mechanisms involved. Additionally, sex hormone levels vary during the menstrual cycle and reliable descriptions of menstrual cycles may be hard to obtain in patients with a severe mental illness. While we controlled for some aspects of antipsychotic drug treatment, other factors may have impacted our results. For example, it is difficult to capture the confounding effects of prior antipsychotic use in cross-sectional studies. Also, due to incomplete data regarding antipsychotic doses and durations of previous drug exposure, we could not calculate cumulative antipsychotic drug exposure. However, in our sample, there were no significant differences between the sexes in regards to being drug-naïve vs. non-naïve or between current antipsychotic users vs. non-users. An additional limitation is that self-reported diet- and exercise-related behaviors may be problematic, as they may be linked to high social desirability. On the other hand, reliable data on diet and exercise may be hard to obtain outside the confinement of an institution. Moreover, we did not assess dietary supplements such as omega-3, which could have influenced our findings related to serum HDL-C and TG levels (86). The average age of our population was also relatively young, and these findings may not necessarily be generalizable to older populations with schizophrenia and related psychoses.

## Strengths and Clinical Implications

To our knowledge, this is the first study in a FEP population that has specifically examined the effect of sex on the relationship between BMI, serum lipid levels with symptoms of psychosis. Moreover, the FEP sample was relatively large and well-characterized, allowing us to adjust our regression models with relevant demographic, lifestyle, illness- and treatment-related factors.

Heterogeneity has been an important challenge in the treatment of schizophrenia, especially in regards to negative symptoms. The findings of the present study may therefore be of clinical relevance as they could argue for more sex-specific usage of serum HDL-C levels for monitoring treatment response in FEP. Both the HDL-C link to negative symptoms and the BMI link to depressive symptoms in females could further indicate a disease mechanism that may be amenable to other interventions, e.g., increasing HDL-C and reducing weight through sex-specific lifestyle modifications and add-on treatments.

## Conclusion

The present study suggests that the relationship between serum HDL-C levels and BMI with specific symptoms of psychosis differs between males and females. Further elucidation of a differential interaction between serum lipids and sex on outcomes, preferentially in longitudinal settings with FEP patients, could help facilitate new therapeutic strategies that are more tailored to the sex of the patient, thus, increasing the likelihood of successful treatment.

## Data Availability Statement

The datasets presented in this article are not readily available because sharing of data to external parties has not been approved by the Ethics Committee. Requests to access the datasets should be directed to prig@norceresearch.no.

## Ethics Statement

The studies involving human participants were reviewed and approved by Regional Committees for Medical and Health Research Ethics, East Norway (REK 1). The patients/participants provided their written informed consent to participate in this study.

## Author Contributions

PG analyzed the data and was responsible for the design of the study, interpretation of results, and drafting of the first version of the manuscript together with IM and VS. OA, CS, TL, and NS contributed with data. All authors contributed to the article and approved the submitted version.

## Funding

This study was supported by grants from the Research Council of Norway to NORMENT CoE (Grant No. 223273/F50, under the Centres of Excellence funding scheme) and Stiftelsen Kristian Gerhard Jebsen (SKGJ-MED-008). The funding bodies had no role in the analyses or writing of the manuscript or the decision to submit this work for publication.

## Conflict of Interest

OA has received speaker's honorarium from Lundbeck and is a consultant for HealhLytix. The remaining authors declare that the research was conducted in the absence of any commercial or financial relationships that could be construed as a potential conflict of interest.

## Publisher's Note

All claims expressed in this article are solely those of the authors and do not necessarily represent those of their affiliated organizations, or those of the publisher, the editors and the reviewers. Any product that may be evaluated in this article, or claim that may be made by its manufacturer, is not guaranteed or endorsed by the publisher.
