# Supplementary material for: Sex-Specific Effect of Serum Lipids and Body Mass Index on Psychotic Symptoms, a Cross-Sectional Study of First-Episode Psychosis Patients
Source: Front Psychiatry. 2021 Oct 21;12:723158. doi: 10.3389/fpsyt.2021.723158 (PMC8566674; doi:10.3389/fpsyt.2021.723158)
Supplement: Supplementary file 1 [file Data_Sheet_1.docx]

**Supplementary table 1.** Antipsychotic medication in the FEP sample

|  | **Males** | | **Females** | | Chi-test | df | p-value |  |
| --- | --- | --- | --- | --- | --- | --- | --- | --- |
|  | N | % | N | % |  |  |  |  |
| **No antipsychotic drug treatment** | 65 | 23 | 37 | 24 | 0.10 | 1 | 0.75 | Antipsychotic user vs. non-users |
| Drug-naïve | 39 | 14 | 26 | 17 | 0.86 | 1 | 0.35 | Drug-naïve vs. non-naïve |
|  |  |  |  |  |  |  |  |  |
| **Antipsychotic monotherapy or polypharmacy** | 218 | 77 | 115 | 76 | 0.64 | 1 | 0.42 | Monotherapy vs. polypharmacy |
| Monotherapy | 166 | 59 | 92 | 61 |  |  |  |  |
| FGA | 4 | 1 | 6 | 4 |  |  |  |  |
| Metabolically potent SGA | 76 | 27 | 30 | 20 | 5.55 | 2 | 0.07 | Metabolically potent vs. intermediate vs. neutral SGA |
| Metabolically intermediate SGA | 56 | 20 | 30 | 20 |  |  |  |  |
| Metabolically neutral SGA | 28 | 10 | 25 | 16 |  |  |  |  |
| Others | 2 | <1 | 1 | <1 |  |  |  |  |
| Polypharmacy with antipsychotics | 52 | 18 | 23 | 15 |  |  |  |  |
| Sum total sample | 283 | 65 | 152 | 35 |  |  |  |  |

FEP= First episode psychosis patients, AP= antipsychotic use, N= number of subjects, %= percentage, df= degrees of freedom, FGA= first generation antipsychotic agents, SGA= second generation antipsychotic agents. For practical purposes we differentiated between three groups of SGA monotherapy based on their metabolic profiles: metabolically potent SGA (focusing on olanzapine), metabolically intermediate SGA (quetiapine and risperidone), and metabolically neutral SGA (aripiprazole, amisulpride, ziprasidone, sertindole).

**Supplementary table 2.** Post-hoc regression analyses examining sex-related associations between serum HDL-C levels and negative symptoms and between BMI and depressive symptoms while adjusting for various covariates.

Only the F-values for the interaction term (sex X metabolic parameter) are listed. The standard beta values in the interaction term are also listed as separate values for males and females.

|  | **PANSS negative factor**    sex X HDL-C interaction | | **CDSS**  sex X BMI interaction | | |
| --- | --- | --- | --- | --- | --- |
|  |  |  |  |  |  |
| Controlling for covariates: | F | B (SE) | F | B (SE) | |
| Diagnosis | F(2,362)=2.84* | M: -1.75 (1.17) | F(2,372)=4.90* | | M: 0.002 (0.07) |
|  |  | F: -1.95 (1.05) * |  | | F: 0.26 (0.08) * |
| Hospitalization | F(2,337)=3.72* | M: -2.16 (1.21) | F(2,346)=6.98* | | M: 0.008 (0.07) |
|  |  | F: -2.21 (1.07) * |  | | F: 0.32(0.09) * |
| Tobacco smoking | F(2,361)=3.18* | M: -1.77 (1.22) | F(2,370)=5.02* | | M: 0.03 (0.07) |
|  |  | F: -2.19 (1.06) * |  | | F: 0.26 (0.002) * |
| Illicit drug use | F(2,320)=3.97* | M: -2.49 (1.31) | F(2,338)=6.02* | | M: 0.08 (0.07) |
|  |  | F: -2.31 (1.11) * |  | | F: 0.29 (0.09) * |
| Prior antipsychotic use | F(2,364)=3.48* | M: -1.86 (1.20) | F(2,338)=6.02* | | M: 0.08 (0.07) |
|  |  | F: -2.27 (1.06) * |  | | F: 0.29 (0.09) * |
| Antipsychotic monotherapy vs. polypharmacy | F(2,372)=3.03* | M: -2.63 (1.50) | F(2,384)=4.74* | | M: 0.07 (0.08) |
|  |  | F: -2.10 (1.22) * |  | | F: 0.29 (0.10) * |
| SGA with different metabolic profiles | F(2,386)=3.39* | M: -2.11 (1.32) | F(2,394)=4.21* | | M: 0.03 (0.07) |
|  |  | F: -2.34 (1.13) * |  | | F: 0.27 (0.09) * |
| Antidepressant use | F(2,364)=3.30* | M: -1.80 (1.20) | F(2,374)=4.60* | | M: 0.007 (0.06) |
|  |  | F: -2.13 (1.06) * |  | | F: 0.24 (0.08) * |

PANSS=Positive and Negative Syndrome Scale, CDSS= Calgary Depression Scale for Schizophrenia, HDL-C= high-density lipoprotein cholesterol, BMI=body mass index, F= f-value, B=regression coefficient, SE= standard error of the regression coefficient, SGA= second generation antipsychotic agents. Illicit drug use was measured with the Drug Use Disorders Identification Test (DUDIT). Prior antipsychotic drug exposure was dichotomized to drug-naïve and non-naïve. For SGA monotherapy we differentiated between three groups of SGA monotherapy based on their metabolic profiles: metabolically potent SGA (focusing on olanzapine), metabolically intermediate SGA (quetiapine and risperidone), and metabolically neutral SGA (aripiprazole, amisulpride, ziprasidone, sertindole). Analyzed with general linear models examining a sex × metabolic parameter (BMI/ HDL-C) interaction controlling for age, sex, and antipsychotic usage (antipsychotic users versus non-users) group.

* p<0.05
